# Evidence for interspecific interactions in the ectoparasite infracommunity of a wild mammal

**DOI:** 10.1186/s13071-016-1342-7

**Published:** 2016-02-02

**Authors:** Sasha Hoffmann, Ivan G. Horak, Nigel C. Bennett, Heike Lutermann

**Affiliations:** Department of Zoology and Entomology, University of Pretoria, Private Bag X20, Hatfield, 0028 South Africa; Department of Veterinary Tropical Diseases, Faculty of Veterinary Science, University of Pretoria, Private Bag X04, Onderstepoort, 0110 South Africa

**Keywords:** Community ecology, Co-infection, Ectoparasite, Interaction, *Rhipicephalus*

## Abstract

**Background:**

Co-infection with multiple parasite species is commonly observed in nature and interspecific interactions are likely to occur in parasite infracommunities. Such interactions may affect the distribution of parasites among hosts but also the response of infracommunities to perturbations. However, the response of infracommunities to perturbations has not been well studied experimentally for ectoparasite communities of small mammal hosts.

**Methods:**

In the current study we used experimental perturbations of the ectoparasite infracommunity of sengis from Africa. We suppressed tick recruitment by applying an acaride and monitored the effects on the ectoparasite community.

**Results:**

Our treatment affected the target as well as two non-target species directly. The experimental removal of the dominant tick (*Rhipicephalus* spp.) resulted in increases in the abundance of chiggers and lice. However, while these effects were short-lived in chiggers, which are questing from the environment, they were long-lasting for lice which spend their entire life-cycle on the host. In addition, the recruitment rates of some ectoparasite species were high and did not always correspond to total burdens observed.

**Conclusion:**

These findings indicate that infracommunity interactions may contribute to patterns of parasite burdens. The divergent responses of species with differing life-history traits suggest that perturbation responses may be affected by parasite life-history and that the ectoparasite infracommunity of sengis may lack resilience to perturbations. The latter observation contrasts with the high resilience reported previously for endoparasite communities and also suggests that anti-parasite treatments can affect the distribution of non-target species.

**Electronic supplementary material:**

The online version of this article (doi:10.1186/s13071-016-1342-7) contains supplementary material, which is available to authorized users.

## Background

The distribution of parasites depends on the exposure and susceptibility of hosts in a population [1, 2]. However, in nature it is rarely a single parasite species that infests an individual host and parasites co-infecting a host can be expected to interact with each other [3, 4]. The nature of such interactions can range from antagonistic to facilitating, depending on whether two parasites interact directly (physically or chemically) or indirectly via shared resources (bottom-up regulation) or the host’s immune system (top-down regulation) [5, 6]. In turn, such interactions may be expected to affect the susceptibility to other parasites and this has implications for disease ecology and epidemiology [7, 8]. Consequently, the infection with one parasite species may reduce or increase the probability of invasion by another parasite species, affect its subsequent establishment and clearance rate and modulate the morbidity and/or mortality and ultimately transmission rates [9, 10].

While interspecific interactions within parasite infracommunities (parasite species assemblage parasitizing a single host) are well documented from laboratory studies [5, 11, 12], similar studies in wild hosts are often correlational and based on cross-sectional data [7, 13–15]. However, recent theoretical and experimental studies have shown that observational approaches may fail to identify interactions between parasites concomitantly infesting a host or incorrectly characterize the type of interaction [16–19]. Consequently, it has been suggested that studies employing experimental manipulations are better suited to identify interspecific relationships between parasite species infesting the same host [18–20].

The resilience (time of recovery from a perturbation) of a community is strongly dependent on the nature and strength of interspecific interactions within a community [21]. These parameters, as well as the complexity of interactions, can in turn determine the effect of perturbations (e.g. removal of a particular species) on the community [21–23]. It has been suggested that these perturbation effects will increase with an increasing number of interactions [21]. In addition, perturbations will result in more long-term effects if they affect keystone species that maintain interactions with many members of the community [24]. However, interspecific interactions and community resilience remain poorly studied in parasite communities and the focus of such studies is biased towards endoparasite communities [7, 13, 14, 17, 19]. Compared to endoparasites our knowledge of the relationships within ectoparasite communities remains limited and largely restricted to within-taxon studies [25–27]. This is despite the complex immunological cascades that some of these ectoparasite species trigger in their hosts [28, 29]. The limited period that the majority of ectoparasitic arthropods spend on a host [30] compared to many endoparasites species may partially account for this bias in the literature. However, ectoparasitic arthropods such as ticks are important vectors for a variety of pathogens of medical and veterinary significance and acaricides are widely used in the livestock and pet industry to reduce tick infestation [28]. Through interspecific interactions the drug-related reduction of tick prevalence and/or abundance could also affect other (non-target) parasite species with potentially important implications for disease ecology.

In the current study we combine observational data and experimental perturbations to study interspecific relationships in the ectoparasite infracommunity of eastern rock sengis (*Elephantulus myurus*) in South Africa. They are small (45–80 g), insectivorous mammals that are widely distributed throughout sub-Saharan Africa [31]. Sengis are one of the dominant species in endemic small mammal communities and sustain a diverse ectoparasite community [32, 33]. The aim of the current study was (i) to assess the prevalence and nature of interactions within their ectoparasite infracommunities, (ii) to determine the exposure of hosts to the various ectoparasite taxa in the environment and (iii) to evaluate the response of the infracommunity to experimental perturbation. We used the acaricide Frontline® (fipronil 10 % w/v/ (s)-methoprene; Merial Pty, Ltd, South Africa) to reduce tick (target parasites) burdens and recruitment. Ticks were chosen as target taxon since they are the most prevalent and abundant ectoparasite taxon sustained by sengis [33]. As haematophagous parasites they not only deplete host resources directly but also trigger well-known immune responses [28]. All of these characteristics make them a likely taxon to interact with several other members of the sengi ectoparasite community either directly or indirectly via resource depletion (i.e. blood) or the immune responses of the host. Consequently, we (i) expected to find evidence for competitive interactions between ticks and other ectoparasite taxa they might physically interact with such as when competing for attachment sites. In addition, we hypothesized that (ii) competitive interactions between ticks and other haematophagous ectoparasites such as lice would be apparent due to competition for resources such as blood.

## Methods

### Collection of animals

Sengis were captured in eight plots at Goro Game Reserve (22°58’S; 29°25’E) in the Limpopo Province, South Africa. Between March 2012 and April 2013 sengis were sampled during six trips (March/April 2012: autumn 12, June 2012: early winter, August 2012: late winter, October 2012: spring, January/February 2013: summer and March/April 2013: autumn 13). Each site was sampled for three nights every second night (Additional file 1: Figure S1) using 150 Sherman traps (H. B. Sherman Traps, Inc. Tallahassee, Florida, U.S.A.) baited with a mixture of sardines, oats and peanut butter. Traps were arranged in 3 × 50 grids with approximately 10 paces between neighbouring traps. Due to the uneven terrain of this grid, this layout had to be adjusted for two of our sites (Additional file 1: Figure S2). Captured individuals were sexed, hand-restrained and all ectoparasites encountered during thorough searches of the entire body were removed with fine-tipped forceps. No ectoparasite eggs were removed during this procedure. The parasites collected were stored in 70 % ethanol for later preparation and identification. Sengis were then given unique ear clips for long-term identification. All animals were released at the point of capture in the afternoon.

### Experimental manipulation

Half of the animals caught were treated against ectoparasites by applying Frontline® with the active component fipronil which kills fleas and ticks. Frontline® was sprayed on the handler’s gloves and then rubbed over the animal’s body as indicated by the supplier. Externally applied it attaches to hair follicles in the dermal skin layer within 24 h but does not reach past the dermis [34]. Topical application of fipronil is effective for over 30 days [35]. The main component of Frontline® spray is isopropyl alcohol which is highly volatile and evaporates within less than 30 min. Fipronil has been successfully applied topically to combat ectoparasite infestations in a number of small mammal species in the wild [36–39]. However, the skin absorption, efficacy and permanency of Fipronil may vary between different species and since such measures were not available for the study species we cannot entirely exclude the possibility that these properties may have slightly deviated for sengis from those reported for other small mammals. Our results (see relevant section) do however, suggest that the treatment was effective. Sengis were randomly assigned to the Frontline® treatment group to achieve an even distribution between the sexes and sites. Once assigned to a treatment group, individuals remained in this treatment group throughout the entire study. The treatment was applied once per trip for each individual irrespective of whether a particular individual was captured for the first time during the first, second or third night on a particular plot during a trip. In addition, an ectoparasite assessment was conducted for each individual during each recapture within the same trip. Effectively, this approach resulted in two treatments: firstly total ectoparasite removal during each capture and secondly manipulation of ectoparasite recruitment rates as a result of the application of Frontline® to half of the individuals captured. However, due to the transient nature of infestation by ectoparasites other than lice [30], the former procedure still allowed an assessment of competitive interactions in the parasite community. At the same time, ectoparasite recruitment rates can serve as a proxy for parasite exposure, a measure notoriously difficult to assess. Mites and lice were cleared and mounted following standard protocols while ticks were identified directly to genus or species-level when possible (for details on identification procedure see [33]). We noted the presence or absence and counted the number of specimens for each parasite taxon using a dissecting microscope.

### Statistical analysis

We calculated the prevalence (proportion of individuals infested) and mean abundance (total number of ectoparasites divided by the number of hosts sampled, Bush et al. [12]) for each parasite taxon. For the analyses we pooled the counts for ticks across stages since the abundance of nymphs was generally too low to permit a meaningful analysis according to life-history stage. Although this was not the case for *Rhipicephalus warburtoni* the abundance of larvae and nymphs was highly correlated (R_S_ = 0.737, *p* < 0.0001) and both stages are present throughout the year [40]. In addition, the qualitative results for the separate stages corresponded to those for the pooled data. Hence only the latter are reported here. Since ticks of the genus *Rhipicephalus* other than *R. warburtoni* had an extremely low prevalence and abundance (see result section) we pooled the data for all specimens from this genus. None of the ectoparasite data collected satisfied the criteria for a normal distribution (Shapiro-Wilk Test: *p* < 0.001) and transformations were unsuccessful. Therefore, we analysed the effect of season, sex, treatment (untreated vs. treated) and capture (see below) on ectoparasite prevalence employing generalised linear mixed models (GLMMs) using a binomial structure with a logit-link function. All two-way interactions were included in the model. To account for repeated sampling of a study plot and individual, the sengi ID nested within site was added as the random effect for all models. GLMMs were run for all ectoparasite taxa exceeding a prevalence of 10 %. Posthoc comparisons were carried out using the least significant difference (LSD). However, few significant effects were found for prevalence and those variables that were significant corresponded to those found for parasite abundance (Additional file 1: Table S1). As a consequence these results are not further discussed. The same variables were included in the full GLMMs for an individual’s ectoparasite abundance but we used a negative binomial data distribution with a log-link function. In addition, we examined the effects of season, treatment, sex and capture on the species richness (number of ectoparasite taxa) of a sengi individual employing GLMMs with a Poisson distribution and a log-link function. The variable sex (either as main effect or interaction term) was not significant for any of the measures examined (p ≤ 0.065) while results changed when it was not included in the models. Hence model results are reported without the terms including sex as a factor.

The only animals captured during October 2012 were five pregnant females. Since pregnancies markedly affect tick burden [41], data for this season were excluded from the analyses. In order to evaluate the overall patterns of parasite distribution as well as the effects of our perturbation experiment we conducted two separate analyses. Firstly, we analysed the data including only the first capture of each individual during each trip (long-term data). We repeated these analyses including recaptures within the same trip to assess short-term patterns (Additional file 1: Figure S1). This allowed us to evaluate the overall evidence for interspecific interactions as well as its effect on recruitment rates. In addition, the time elapsed between consecutive trips (8–12 weeks) exceeded the period indicated by the manufacturer for the effectiveness of Frontline®. Thus analysing data for short-time intervals were conducted to evaluate the effectiveness of our treatment. For these short-term analyses, capture indicated that animals were captured for the first, second or third time within a trip. When analysing long-term effects individuals were classified as either new animals when first captured during the course of this study or as recaptures in a subsequent trip (i.e. capture for analyses of long-term data). For the short-term data analyses that included recaptures within the same trip the variable ‘capture’ had three levels for the first, second or third time that an individual was caught within a particular trip. Models were simplified using backward stepwise elimination of non-significant terms beginning with interaction terms to obtain the minimal model. We evaluated the validity of the final model by comparing the results from this approach with those based on model selection based on the Akaike information criterion [42]. To evaluate the contribution of our random factors we used general linear models using the same data distributions and variables as indicated above. However, since with few exceptions (e.g. short-term abundance of *Rhipicephalus* spp.) omitting the random effects changed the qualitative results of the models. Consequently, we are only reporting the results for GLMMs. All analyses were carried out in SPSS v.22 and results are reported as means ± standard error (SE).

## Results

### Ectoparasite burdens

A total of 125 animals were caught (68 ♂, 57 ♀) between one and ten times during the study period (333 captures in total). Nine tick species, belonging to six genera*,* one mite and one louse species were collected from these animals (Table 1) with individuals harbouring between 0 and 5 parasite species at a time (Additional file 1: Figure S3). Only immature ticks (i.e. larvae and nymphs) were recovered from the animals. Unlike reported for other sengi populations [33, 43], no fleas were found in the study population during the course of the study. Only two of the ticks (*Rhipicephalus* (*R*.) *warburtoni* and *Rhipicentor* (*Rc*.) *nuttalli*), chigger mites and the louse *Neolinognathus elephantuli* occurred at substantial prevalence or abundance with *R. warburtoni* being the most prevalent (100 % of first captures) and abundant (mean abundance per individual: 259.02 ± 129.03 for first captures only). With the exception of the mite all of these species prefer sengis as hosts [33]. In contrast, chiggers are host generalists and exploit a wide range of hosts [44].

**Table 1:** Ectoparasite species collected and their infestation parameters on *Elephantulus myurus* in the Goro Game Reserve

Taxon	Species	Total	Prevalence [%] (95 % CI)	Abundance (95 % CI)
Ticks	*Argas brumpti*	3	0.6 (0.001 – 0.023)	0.01 (0.00–0.02)
	*Amblyoma hebraeum*	8	0.5 (0.00 – 0.03)	0.04 (0.00 – 0.11)
	*Amblyoma marmoreum*	5	2.3 (0.01 – 0.05)	0.02 (0.00 – 0.05)
	*Haemaphysalis elliptica*	5	1.2 (0.00 – 0.04)	0.01 (0.00 – 0.02)
	*Nuttalliella namaqua*	32	0.3 (0.00 – 0.02)	0.10 (0.00 – 0.29)
	*Rhipicentor nuttalli*	987	48.3 (0.43 – 0.54)	3.11 (2.49–3.75)
	*Rhipicephalus arnoldi*	2	0.9 (0.00 – 0.03)	0.01 (0.00–0.02)
	*Rhipicephalus simus*	11	3.3 (0.01 – 0.07)	0.05 (0.02 – 0.09)
	*Rhipicephalus warburtoni*	54187	94.9 (0.92 – 0.97)	169.80 (152.44 – 188.52)
Mites	Trombiculidae (chiggers)	2713	66.2 (0.60 – 0.73)	15.30 (11.97 – 19.74)
Lice	*Neolinognathus elephantuli*	928	15 (0.07 – 0.14)	2.77 (1.80 – 4.06)

### Treatment effects on parasite distribution

**Fig. 1:**
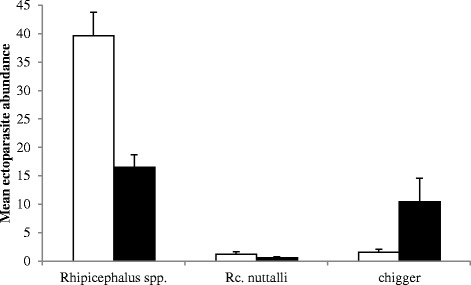
Effects of treatment on the abundance of *Rhipicephalus* spp., *Rc. nuttalli* and chiggers when all capture data are considered. Untreated animals are indicated with open bars while solid bars indicate treated animals. Displayed are means ± SE

Lice were the only ectoparasite taxon affected by our treatment when only long-term data were considered and treated animals (14.8 ± 7.9) sustained a significantly greater abundance than untreated individuals (2.3 ± 1.2, Table 2). In contrast, all ectoparasite taxa except lice showed a significant effect of treatment on their abundance when short-term data were considered (Table 2). While the abundance of the two tick taxa was significantly reduced in treated compared to untreated individuals, the opposite was true for chiggers (Fig. 1). When considering short-term but not long-term data the treatment resulted in a significantly reduced parasite species richness in treated (1.57 ± 0.15) compared to untreated individuals (1.34 ± 0.14, Table 3).

**Table 2:** Results of the final GLMMs for long- and short-term effects of season, treatment and capture on the ectoparasite abundance of *E. myurus*

	Variable	*Rhipicephalus spp.*		*Rc. nuttalli*		chigger		*N. elephantuli*	
		*F*-value	*p*-value	*F*-value	*p*-value	*F*-value	*p*-value	*F*-value	*p*-value
Long-term data	Season	5.813	**<0.0001**	2.367	0.054	5.690	**<0.0001**	0.223	0.925
	Treatment	-	**-**	0.000	0.998	0.581	0.447	9.057	**0.003**
	Capture	-	**-**	0.000	0.999	0.077	0. 781	0.793	0.374
	Season*treatment	-	-	0.720	0.579	1.371	0.246	0.562	0.690
	Season*capture	-	-	0.156	0.926	2.520	0.059	0.297	0.827
	Treatment*capture	-	**-**	0.744	0.390	0.344	0.558	2.081	0.151
Short-term data	Season	7.213	**<0.0001**	4.104	**0.003**	0.781	0.538	0.555	0.696
	Treatment	30.678	**<0.0001**	6.911	**0.009**	13.711	**<0.0001**	1.261	0.262
	Capture	447.649	**<0.0001**	9.562	**<0.0001**	9.065	**<0.0001**	4.433	**0.013**
	Season*treatment	1.654	0.161	-	-	0.547	0.702	0.277	0.893
	Season*capture	5.524	**<0.0001**	0.560	0.810	0.348	0.946	0.206	0.990
	Treatment*capture	30.089	**<0.0001**	3.731	**0.025**	9.305	**<0.0001**	3.488	**0.032**

-: factor dropped from the final model

Significant effects are highlighted in bold

**Table 3:** Results of the final GLMMs for long- and short-term effects of season, treatment, capture status, sex on the ectoparasite species richness of *E. myurus*

Variable	Long-term		Short-term	
	*F*-value	*p*-value	*F*-value	*p*-value
Season	4.216	**0.003**	2.115	0.079
Treatment	-	-	4.013	**0.046**
Capture	-	-	11.450	**<0.0001**
Season*treatment	-	-	0.284	0.971
Season*capture	-	-	-	-
Treatment*capture	-	-	-	-

-: factor dropped from the final model

Significant effects are highlighted in bold

### Treatment effects on ectoparasite recruitment rates

Although capture had no significant effect on long-term ectoparasite abundance, it significantly affected all four ectoparasite taxa as well as species richness when short-term data were considered (Tables 2 and 3). For all taxa abundance was significantly higher during the first compared to the second and third capture (LSD: p ≤ 0.050, Additional file 1: Figure S4). In addition, it decreased significantly from second to third capture for both ticks (LSD: p ≤ 0.018, Additional file 1: Figure S4) but none of the other taxa or species richness (LSD: p ≥ 0.471). Similarly, species richness was significantly lower during the second (1.2 ± 0.1, LSD: *p* < 0.0001) and third (1.2 ± 0.3, LSD: *p* = 0.006) compared to the first capture (2.1 ± 0.1, Table 3) while it did not differ significantly between second and third capture (LSD: *p* = 0.891).

**Fig. 2:**
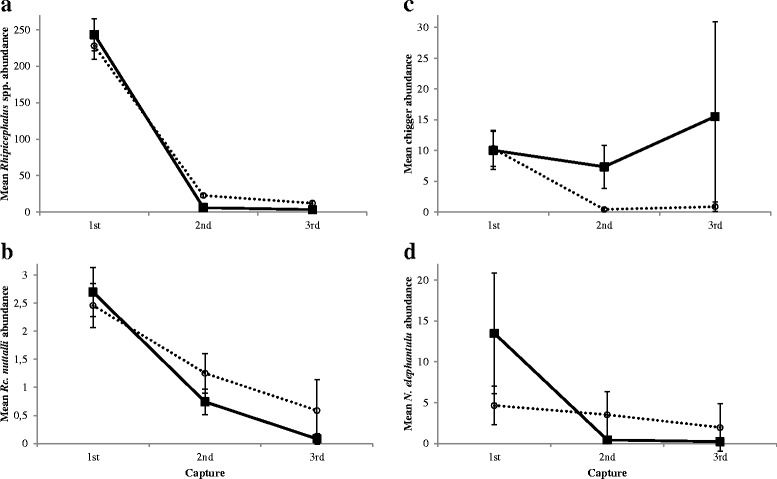
Effects of capture (first (*n* = 205), second (*n* = 99) and third (*n* = 29) of an individual within a trip) and treatment on the abundance of (**a**) *Rhipicephalus* spp., (**b**) *Rc. nuttalli*, (**c**) chiggers and (**d**) *N. elephantuli*. Untreated animals are depicted with dotted lines and open circles while treated individuals are represented with filled squares and solid lines. Displayed are means ± SE

For short-term data the interaction between treatment and capture was significant for all four ectoparasite taxa (Fig. 2, Table 1). The abundance during the first capture did not differ significantly between treatments for any of the ectoparasite taxa (LSD: p ≥ 0.255). In contrast, the mean abundance of *Rhipicephalus* spp. was significantly lower for treated than untreated sengis for both second and third captures (LSD: p ≤ 0.0002, Fig. 2a). However, mean abundance did not differ significantly between treatments for the second and third capture for *Rc. nuttalli* and lice (p ≤ 0.148, Fig. 2b and d). Conversely, the mean chigger abundance was significantly greater for treated compared to untreated animals for second (LSD: *p* = 0.046) but not third captures (LSD: *p* = 0.340, Fig. 2c). At the same time, among treated individuals abundance differed significantly between all captures for both tick species (LSD: p ≤ 0.021, Fig. 2a and b). Similarly, the mean abundance of *N. elephantuli* was significantly greater during the first capture compared to both second and third capture (LSD: p ≤ 0.050, Fig. 2d) but not between second and third capture (LSD: *p* = 0.706) for treated sengis. In contrast, the mean abundance of chiggers did not differ significantly between captures (LSD: p ≥ 0.554) of treated individuals. Among untreated sengis, the mean abundance of *Rhipicephalus* spp. decreased significantly between successive captures (LSD: p ≤ 0.006, Fig. 2a) while it was significantly lower during second and third capture compared to the first capture for *Rc. nuttalli* and chiggers (LSD: p ≤ 0.021; Fig. 2b and c) but did not differ significantly between the last two captures (LSD: p ≥ 0.306). In contrast, mean *N. elephantuli* abundance did not vary significantly between captures for untreated individuals (p ≥ 0.470).

### Other factors affecting ectoparasite abundance

**Fig. 3:**
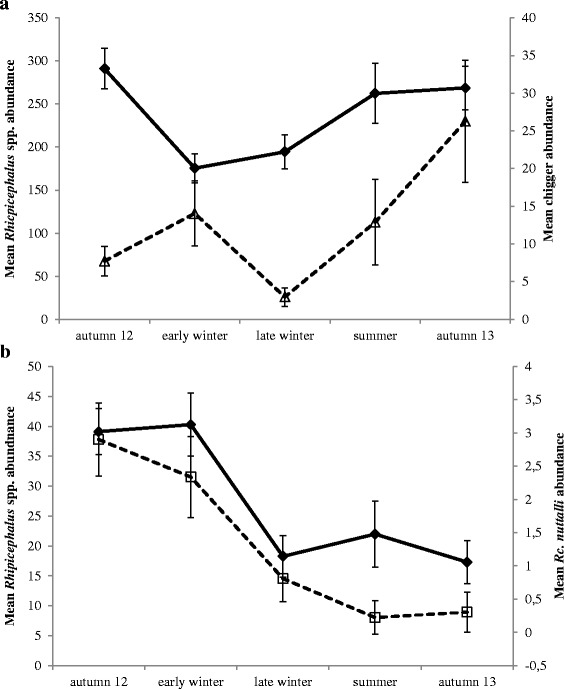
Seasonal variation in (**a**) the long-term abundance of *Rhipicephalus* spp. (black line, filled diamonds) and chiggers (dashed line, open triangles) and (**b**) the short-term abundance of *Rhipicephalus* spp. and *Rc. nuttalli* (dashed line, open squares) sustained by *E. myurus* during the study period. Displayed are means ± SE

The mean long-term abundance of *Rhipicephalus* spp. and chiggers but not of the other two species differed significantly between seasons (Table 2). For *Rhipicephalus* spp. it was significantly lower in early winter compared to all other seasons (LSD: p ≤ 0.025, Fig. 3a) except late winter (LSD: *p* = 0.453). Similarly, the mean abundance of *Rhipicephalus* spp. in late winter was significantly lower than during autumn 12 and autumn 13 (LSD: p ≤ 0.024, Fig. 3a) but not summer (LSD: *p* = 0.089). No other pairwise comparisons were significant. The seasonal variation in chigger abundance differed from that of *Rhipicephalus* spp. and was significantly lower in late winter compared to all other seasons (LSD: p ≤ 0.042) except summer (LSD: *p* = 0.089, Fig. 3a). In addition, it was significantly greater in autumn 13 compared to autumn 12 (LSD: *p* = 0.027, Fig. 3a) while no other pairwise comparisons were significant (LSD: p ≥ 0.089). However, the long-term species richness differed significantly between seasons (Table 3). It was significantly lower in late winter and spring compared to the other seasons (LSD: p ≤ 0.042, Additional file 1: Figure S2) while none of the remaining pairwise comparisons were significant (LSD: p ≥ 0.388).

Season significantly affected the short-term abundance of both ticks but not chiggers and lice (Fig. 3b, Table 2). For both ticks mean abundance was significantly greater during autumn 12 and early winter compared to the other seasons (LSD: p ≤ 0.026, Fig. 3b). None of the remaining pairwise comparisons was significant (LSD: p ≥ 0.170).

For short-term data the interaction between season and capture was significant for the abundance of *Rhipicephalus* spp. (Table 2). It was significantly higher for first compared to both second and third captures during all seasons (LSD: *p* < 0.0001 for all, Additional file 1: Figure S6). In contrast, it was significantly lower for third compared to second captures in autumn 12 and late winter (LSD: p ≤ 0.029, Additional file 1: Figure S6) but not the other seasons (LSD: p ≥ 0.117). In addition, it was significantly lower in early winter compared to autumn 12 and 13 (LSD: p ≤ 0.019) and late winter compared to autumn 12 (LSD: *p* = 0.017, Additional file 1: Figure S6) for first captures. Conversely, it was significantly higher in autumn 12 and early winter compared to all other seasons for second captures (LSD: p ≤ 0.010, Additional file 1: Figure S6) while no other pairwise comparisons were significant. Among third captures the abundance of *Rhipicephalus* spp. was significantly lower in late winter compared to autumn 12 and early winter (LSD: p ≤ 0.014, Additional file 1: Figure S6). In addition, it was significantly lower in summer compared to autumn 12 (LSD: *p* = 0.011) and early winter compared to autumn 12 (LSD: *p* = 0.015, Additional file 1: Figure S6), while no other pairwise comparison was significant (LSD: p ≥ 0.063).

## Discussion

Using a combination of observational data and experimental manipulation we provide one of the first studies demonstrating interspecific interactions within the ectoparasite community of a small mammal. Similar to what has previously been reported for endoparasite communities of small mammals [17, 19] we found evidence for competitive interactions between parasite taxa. Furthermore, our data indicate high recruitment rates for the two most prevalent and abundant ectoparasite species suggesting high host exposure. This contrasts strongly with the findings of another recent study on sengis that reported low recruitment rates for the same two ectoparasite species [43]. A number of reasons may account for these differences. Firstly, the sampling regimen differed slightly between both studies with only about half the number of traps used in [43] and traps being opened over four consecutive nights. Also, the study site of the current study and the locality studied in [43], which is located ca. 300 km south in South Africa’s Gauteng province, differ vastly with regards to climate, vegetation cover and small mammal community composition with Goro Game Reserve having a much hotter and drier climate with a continuous rocky landscape and scarce vegetation cover and a less diverse small mammal community comprised of four species (three rodents: *Micaelamys namaquensis*, *Aethomys chrysopilus* and *Acomys spinossissimus*; and the study species) [40, 45]. In contrast, at the Gauteng study site rocky outcrops are interspersed in grassland or savannah habitat and the small mammal community is much more diverse (nine rodents and two insectivores including the study species [32]. For host specialists such as *R. warburtoni*, the lower abundance of sengis may account for the observed differences between the studies. However, this cannot account for the differences in chigger recruitment and although the prevalence of chiggers was comparable in both sites it was almost one order of magnitude lower in the current study compared to [43]. Unlike *R. warburtoni* chiggers are not arid specialists and the lack of vegetation cover may result in a more severe risk of desiccation in our study site. At the same time, the greater host diversity in the Gauteng site may sustain larger numbers of this generalist parasite.

### Evidence for competitive interactions and possible mechanisms mediating competition

We found that treated sengis exhibited an almost four-fold reduction in *Rhipicephalus* spp. recruitment that coincided with an18-fold increase in chigger recruitment immediately following treatment with an acaricide. At the same time, despite the lack of long-term treatment effects on *Rhipicephalus* spp. the abundance of lice increased more than six-fold over the study period in treated but not untreated individuals suggesting a between-taxa competition. For long-term data the species richness was lowest when the abundance of *Rhipicephalus* spp. was greatest corroborating the hypothesis that dominant or keystone species strongly influence ecological communities [24].

The different temporal patterns observed for chiggers and lice, respectively, suggest that the mechanisms mediating the competitive relationship with *Rhipicephalus* spp. differs for the two taxa. Since both ticks and *N. elephantuli* are haematophagous, indirect competition for resources could account for the increase in louse burden [6]. However, competition for resources is unlikely to explain the relationship between these ticks and chiggers since chiggers feed on liquefied epithelial cells and tissue [44]. Thus, the high abundance and prevalence of *Rhipicephalus* spp. and chiggers could suggest a direct competition for attachment sites. The vast majority of *Rhipicephalus* spp. were *R. warburtoni* and sengis are their preferred host [33]. On sengis this tick is predominately found on the ridges of the ears and the base of the lower back while chiggers are attached to the lower back only. In contrast, in sympatric rodents, where *R. warburtoni* is absent, chiggers are usually found on the ears [43] supporting this hypothesis. Similar competition for attachment sites between ticks and mites has been suggested for *Ixodes pacificus* and chiggers parasitizing lizards [46] further corroborating our hypothesis. Since competition for attachment sites has been reported between co-infecting ticks species [25, 26] and our treatment would have targeted all tick species, we cannot entirely exclude the possibility that such a competitive relationship may also exist between *Rhipicephalus* spp. and *Rc. nuttalli*. At the same time, the more than 30 times greater abundance of *Rhipicephalus* spp. compared to *Rc. nuttalli* makes it unlikely that the removal of *Rc. nuttalli* alone would have resulted in similar increases of chiggers and lice.

Evidence for a strong competitive interaction between the dominant tick species and chiggers exploiting sengis has previously been reported for the study species and thus appears to be consistent regardless of differences in climate, host density or small mammal community composition. In contrast, evidence for interspecific interactions between co-infecting ectoparasite species was reported in the Gauteng sengi population with prevalences or abundances of several less prevalent and/or abundant tick species increasing as a result of the treatment while others appear to respond to other ectoparasite taxa. At the same time, although lice were found in the Gauteng population no long-term treatment effects such as reported in the current study were found [43]. This may be partially linked to the more diverse ectoparasite community recorded for the more southern sengi population that comprises at least eleven tick species of which four exceed prevalences of 15 %, chiggers, *N. elephantuli* as well as five flea species [43]. This may also account for the differences in the distribution of certain ectoparasite taxa and although the prevalence and abundance of the dominant tick species were comparable between both populations this did not apply to the remaining most common ectoparasite taxa found in the current study. In addition, the previous study was conducted over a longer time period (3 years) than the current study and may thus have been able to pick up additional, more subtle relationships such as those between rarer tick species. It furthermore suggests that caution should be applied when attempting to generalize the findings from one host population to others.

### The role of parasite life-history traits in interspecific competition within the ectoparasite community

The contrasting temporal patterns in the competitive interactions between *Rhipicephalus* spp. and lice and chiggers, respectively, could be linked to the differences in host exposure to *N. elephantuli* and chiggers as well as differences in life-history traits between these two species. While only the larval stage of chiggers are parasitic and our recruitment data suggest that they are present in the environment throughout the year, lice spend their entire life-cycle on the host [30, 44]. Consequently, chigger recruitment would largely be determined environmentally, while the recruitment of lice depended mostly on lice hatched from the eggs that remained on the sengis after each capture [30, 47]. Given the extremely high abundance of *R. warburtoni* among first captures, a substantially reduced feeding competition could be expected for several weeks as a result of our treatment. This is likely to have led to greater reproductive success once these lice reached the adult stage and could account for the overall increase in *N. elephantuli* abundance. However, unlike with the permanently questing chigger larvae present in the environment, the developmental time meant that this effect would only be apparent with a certain time delay.

### Variables affecting community responses to perturbations

It has been suggested that the nature of interspecific interactions determines the resilience of a community [21–23]. Our finding that the duration of treatment effects differed between ectoparasite taxa which compete via different mechanisms (i.e. direct vs. indirect) provides corroborating evidence for this hypothesis. In addition, chiggers and lice differ markedly in their life-history traits and this suggests that variation in life-history strategies of non-target members of the parasite community should be taken into account when targeted treatment is considered for management purposes. The importance of such context-dependent approaches to avoid undesirable infracommunity responses has previously been highlighted for the epidemiology of endoparasite communities [9, 10, 17].

Although our results suggest competitive interactions between the most common and abundant ectoparasite, *Rhipicephalus* spp., for both chiggers and lice the divergent temporal responses of these two species to the removal of *Rhipicephalus* spp. do not lend support to the hypothesis that community resilience will be lower if perturbations affect keystone or dominant species with many interactions in the community [24]. Instead the mechanisms governing resilience appear to be more complex for ectoparasite communities and both life-history as well as interaction mechanism may contribute to the community response to the removal of dominant species.

### Contributions of other factors to the distribution of ectoparasites

Our analyses revealed seasonal fluctuations for ticks and chiggers that quest from the environment. This is in accordance with the notion that ectoparasites that spend a large proportion of their life-cycle off-host are generally assumed to be predominately affected by environmental rather than host factors [30, 40, 48, 49]. In contrast, the lack of seasonal effects observed for *N. elephantuli* are probably a result of the fact that lice spend their entire life-cycle on the host and are thus more dependent on ‘host microclimate’ than environmental factors [30, 47]. However, seasonal differences in recruitment rates were only apparent for *Rhipicephalus* spp. suggesting that apart from environmental and host factors [40, 41] interspecific interactions within the infracommunity may play a role in generating the seasonal patterns observed in this and other studies. This hypothesis deserves further attention in future studies.

## Conclusion

We found evidence for several competitive interspecific interactions between members of the ectoparasite infracommunity of sengis. However, the competitive mechanisms mediating these interactions are likely to differ between taxa. In addition, long-term effects of our experimental perturbation were observed for lice, while they were only of short duration for chiggers indicating that in addition to the nature of community interactions, parasite life-history may affect community resilience. It furthermore suggests that the application of common anti-parasite treatments targeting particular parasite groups can also affect non-target parasites.

### Ethical approval

This research was conducted under permit number 001-CPM401-0002 from the Department of Environmental Affairs, Limpopo Province, Africa and approved by the Ethics committee of the University of Pretoria (EC11-12).

## Additional file

Additional file 1: **Supplementary material.** Evidence for interspecific interactions in the ectoparasite infracommunity of a wild mammal. (PDF 646 kb)
