# Supplementary material for: Evidence for interspecific interactions in the ectoparasite infracommunity of a wild mammal
Source: Parasit Vectors. 2016 Feb 2;9:58. doi: 10.1186/s13071-016-1342-7 (PMC4735965; doi:10.1186/s13071-016-1342-7)
Supplement: Supplementary file 1 — Supplementary material. Evidence for interspecific interactions in the ectoparasite infracommunity of a wild mammal. (PDF 646 kb) [file 13071_2016_1342_MOESM1_ESM.pdf]

Additional file 1: Evidence for interspecific interactions in the ectoparasite infracommunity of a wild mammal.

Sasha Hoffmann, Ivan G. Horak, Nigel C. Bennett and Heike Lutermann*

*Department of Zoology & Entomology, University of Pretoria, Email: hlutermann@zoology.up.ac.za

Figure S1. Outline of the trapping schedule used on all eight study plots. For each plot 150 Sherman traps were brought every second night (indicated by the arrows) during each of the five trips (autumn 12, early and late winter, summer and autumn 13) conducted. Numbers below the arrows indicate the number of individuals caught as first (bold arrows), second (broken line arrow) and third (dashed arrows) during each trip. Only the individuals caught as first captures were used for the long-term analyses while all captures were included for short-term patterns.

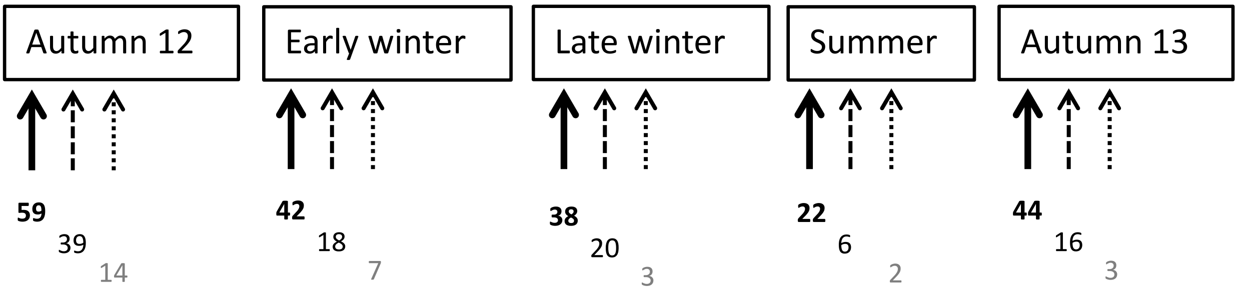

Figure S2. Spatial distribution of the eight study plots sampled during the current study. Grey dashed lines indicate the trap lines (50 traps each). Plots were only accessible via a nearby road and the uneven terrain required and adjustment of the trapping grid layout for site 3 and 8.

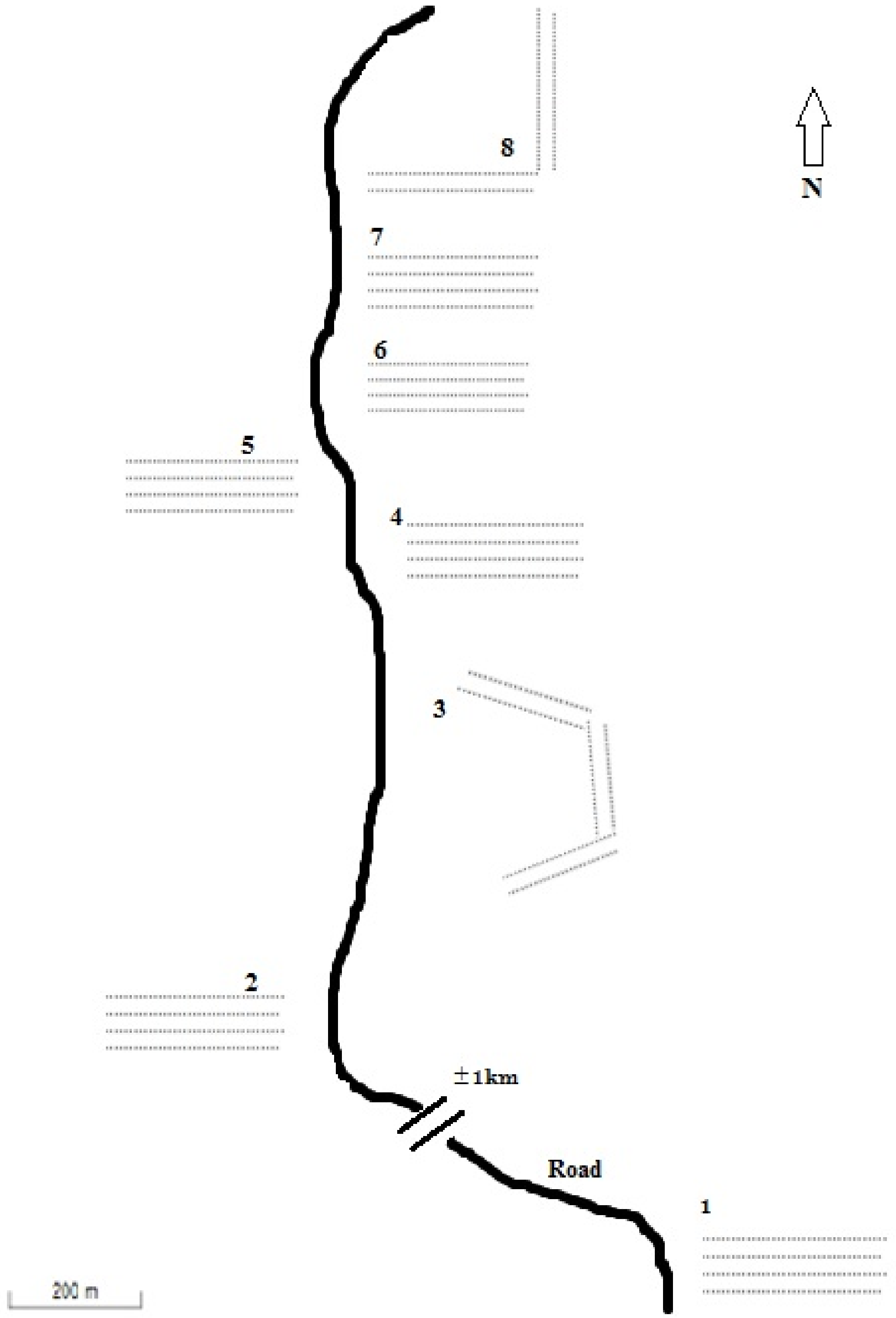

Figure S3. Distribution of the number of co-infecting ectoparasite species for first captures only (open bars) and all captures (solid bars) during the entire study period.

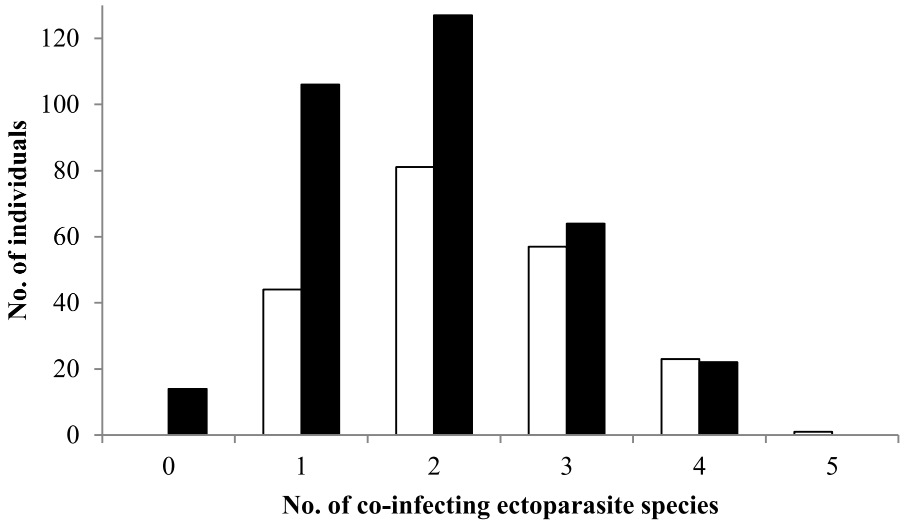

Figure S4. Effects of capture (first, second or third within the same trip) on the abundance of the four main ectoparasite taxa sustained by *E. myurus*.

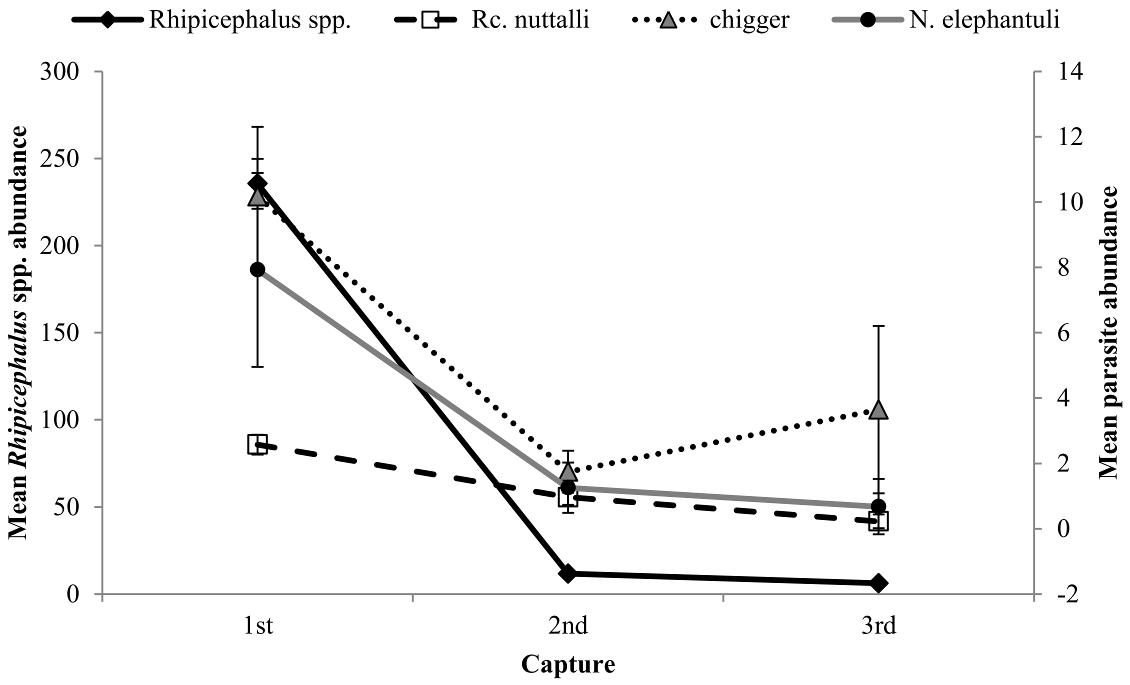

Figure S5. Seasonal variation in ectoparasite species richness maintained by *E. myurus* during the study period.

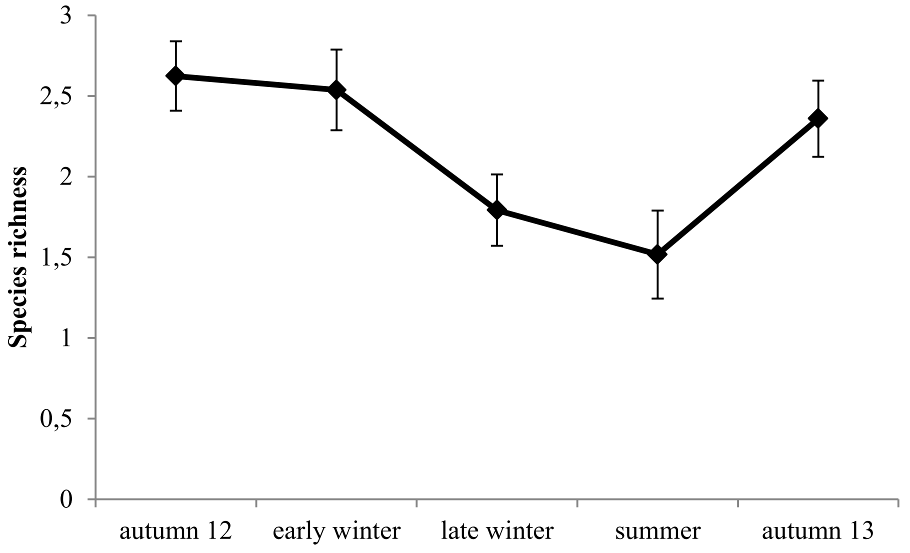

Figure S6. Seasonal variation in the abundance of *Rhipicephalus* spp. during the first (solid line, solid squares), second (dashed line, grey squares) and third capture (dotted line, open circles) of *E. myurus* during the study period.

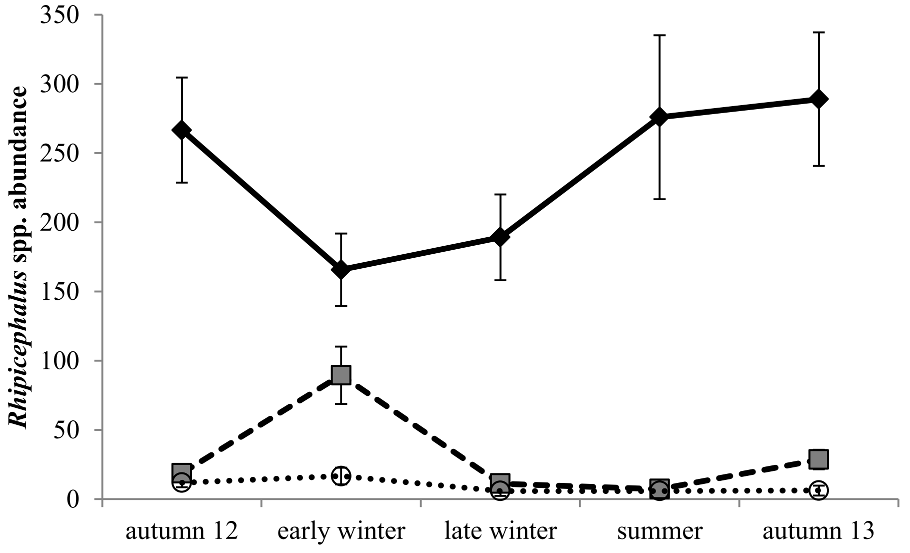

Table S1. Results of the final GLMMs for long- and short-term effects of season, treatment, capture status, sex on the ectoparasite prevalence of *E. myurus*. Significant effects are highlighted in bold. Note that prevalence was 100% for *Rhipicephalus* spp. among first captures.

	Variable	***Rhipicephalus spp.***		***Rc. nuttalli***		chigger		***N. elephantuli***	
		F-value	p-value	F-value	p-value	F-value	p-value	F-value	p-value
**Long-term data**	Season	-	**-**	2.897	**0.023**	4.755	**0.001**	0.350	0.844
	Treatment	-	**-**	0.042	0.838	0.002	0.965	0.000	1.000
	Capture	-	**-**	0.000	1.000	1.760	0. 186	0.000	1.000
	Season*treatment	-	-	-	-	-	-	0.450	0.773
	Season*capture	-	-	1.108	0.347	0.745	0.527	0.528	0.664
	Treatment*capture	-	**-**	0.310	0.589	1.999	0.159	0.216	0.643
**Short-term data**	Season	0.476	0.754	1.154	0.331	6.733	**<0.0001**	1.243	0.293
	Treatment	1.342	0.248	7.843	**0.005**	0.572	0.450	0.000	0.999
	Capture	3.787	**0.024**	0.000	1.000	24.144	**<0.0001**	5.800	**0.003**
	Season*treatment	0.082	0.988	-	-	-	-	0.271	0.890
	Season*capture	0.429	0.903	0.247	0.981	-	-	-	-
	Treatment*capture	1.484	0.228	4.696	**0.010**	0.097	0.908	-	-

-: factor dropped from the final model.
